# Primary peripheral T-cell lymphoma of the cervix with mononeuritis multiplex: an unusual case presentation

**DOI:** 10.4322/acr.2021.354

**Published:** 2022-02-11

**Authors:** Ratul Seal, Mayur Parkhi, Rajesh Kumar, Suvradeep Mitra

**Affiliations:** 1 All India Institute of Medical Sciences, Department of General Medicine, Bhubaneswar, India; 2 Post Graduate Institute and Medical Education and Research (PGIMER), Department of Histopathology, Chandigarh, India; 3 All India Institute of Medical Sciences, Department of Pathology and Laboratory Medicine, Bhubaneswar, India

**Keywords:** Lymphoma, T-Cell, Peripheral, Cervix Uteri, Mononeuropathies, Cerebrospinal Fluid, Immunohistochemistry

## Abstract

Peripheral neuropathy (PN) is characterized by the injury to the peripheral nervous system of varied etiology. Lymphoma is one of the etiologies of PN, presenting various neurological manifestations. Neuropathy associated with peripheral T-cell lymphoma, not otherwise specified (PTCL, NOS) is unusual and fewer cases are documented in the literature. In addition, PTCL, NOS is extremely rare as primary in the female genital tract, especially uterine cervix, and exhibits aggressive clinical course with poor therapy response. We hereby describe a 47-year-old female who presented with fever and chills for 15 days. Clinical examination revealed left-sided lower motor neuron type of facial nerve palsy with Bell’s phenomenon. Nerve conduction study of all four limbs illustrated asymmetrical axonal neuropathy (motor > sensory), suggesting mononeuritis multiplex. She developed vaginal bleeding during her hospital stay. Pelvic examination and imaging revealed a 4x3cm polypoidal mass on the posterior lip of the cervix, which was excised and diagnosed as extranodal primary PTCL, NOS based on morphology, immunohistochemistry, and in-situ hybridization findings. Besides, the cerebrospinal fluid (CSF) was infiltrated by the lymphoma cells, detected on cell block preparation. The patient succumbed to her illness within one week despite best efforts and the commencement of chemotherapy. No consent was obtainable for nerve biopsy and autopsy. Thus, we report an extremely rare case of primary extranodal PTCL, NOS of the uterine cervix with unusual presentation of mononeuritis multiplex. Further, we discussed the differentials of PTCL, NOS at this extranodal site.

## INTRODUCTION

Peripheral T-cell lymphoma, not otherwise specified (PTCL, NOS), is a mature T-cell lymphoma that accounts for approximately 30% of PTCL in western countries.^1^ This entity represents heterogeneous neoplasms diagnosed by excluding other specific entities tabulated under mature T- and NK-cell neoplasms in the 2017 edition of the World Health Organization (WHO) classification.^1^ PTCL, NOS mostly have peripheral lymph node involvement; however, extranodal sites such as liver, spleen, bone marrow, gastrointestinal tract, and skin can also be involved.^1^ The female genital tract (FGT), especially the cervix as primary extranodal site, is extremely rare as the reported ones represent mainly B-cell lymphoma, i.e. diffuse large B-cell lymphoma.^2^^,^ 3 Peripheral neuropathy is associated with malignant lymphoma in merely 5-8% of cases.^4^ Fewer case reports of PTCL in association with neuropathy are documented in the English literature.^5^^,^ 6 Hereby, we discuss a female patient who presented with mononeuritis multiplex and cervical polypoidal mass, later diagnosed as PTCL, NOS on biopsy. The index case with primary T-cell lymphoma at an uncommon site with unusual clinical presentations is worthy of discussion and documentation.

## CASE REPORT

A 47-year-old lady presented to the emergency services of our institute with fever and chills for 15 days, following which she was admitted. For the last one day, it was associated with burning pain and weakness in the left upper limb, and right-side deviation of the angle of mouth. No significant past history was obtained. Clinical examination revealed a left sided lower motor neuron (LMN) type of facial nerve palsy accompanying with Bell’s phenomenon. Motor system examination showed decreased power in the left upper limb [shoulder joint (flexion and extension 2/5; abduction 2/5; adduction 3/5) and elbow joint (extension 4/5; flexion 2/5)] with sparing of the wrist and small joints of the hand. There were diminished deep tendon reflexes on the left-sided biceps, triceps, and supinator, representing clinically an LMN type of weakness. Nerve conduction study of all four limbs illustrated asymmetrical axonal neuropathy (motor > sensory), suggestive of mononeuritis multiplex. The affected motor nerves were left ulnar, median, and right peroneal. Involved sensory nerves included left ulnar and right superficial peroneal. Nerve biopsy was not performed as consent from the patient could not be obtained. MRI-brain showed non-specific small white matter hyperintensities in multiple areas of the brain. Cerebrospinal fluid (CSF) analysis showed mildly elevated protein levels with lymphocytic pleocytosis. Routine investigations revealed leukopenia (total leukocyte count - 2.1 x 10^-9^/L; RR: 3.9 to 11.1 x 10^-9^/L) with relatively normal differential count (neutrophils 1.1 x 10^-9^/L [RR: 1.7-7.5 x 10^-9^/L], lymphocytes 0.7 x 10^-9^/L [RR: 1.0 - 3.2 x 10^-9^/L], monocytes 0.1 x 10^-9^/L [RR: 0.2-0.6 x 10^-9^/L], eosinophils 0.2 x 10^-9^/L [RR: 0.03-0.46 x 10^-9^/L]) while the erythrocyte sedimentation rate and C-reactive protein were within normal range. Serum lactate dehydrogenase level was elevated (633.5 U/L; RR: 140 to 280 U/L). The fasting serum glucose, renal function test, autoimmune autoantibody profile, and serum electrophoresis were normal. Mild elevation (1.5-2 times) of the transaminases was noted. The ANCA serology workup was within normal limits. The serologies for HIV, hepatitis B, hepatitis C, EBV, and CMV were non-reactive. She developed vaginal bleeding during her hospital stay. Surprisingly, a pelvic examination revealed a polypoid growth on the cervix’s posterior lip of size 4x3cm, which bled on touch when examined. Ultrasonography (USG) and contrast-enhancing computed tomography (CECT) of the abdomen confirmed the bulkiness of the cervix, although no lymphadenopathy or solid organ deposits were noted in the patient. Patient was not subjected to the PET scan imaging.

**Figure 1:**
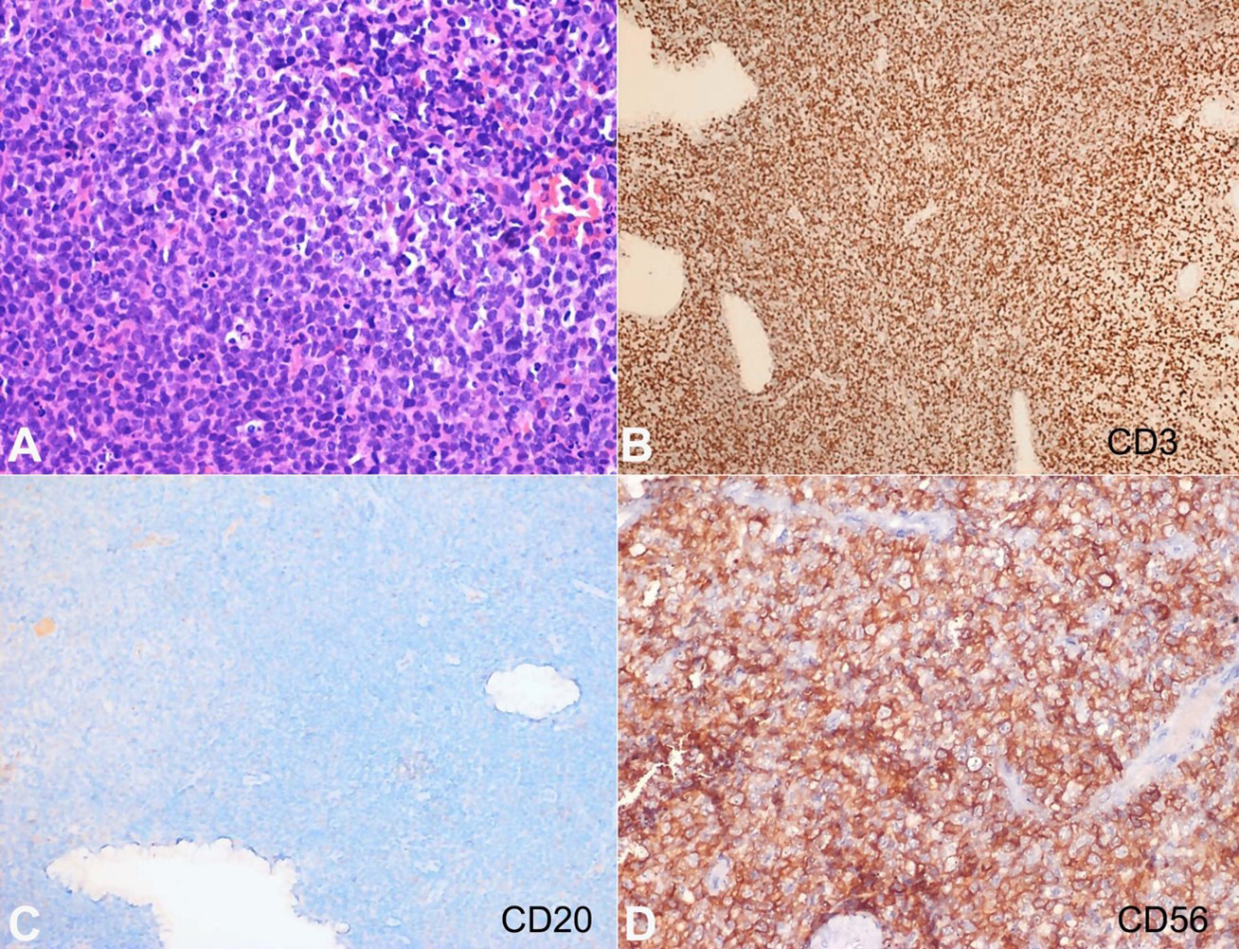
Photomicrographs of the cervical tumor. **A –** Diffuse sheets of atypical lymphoid cells having small-to-medium size morphology (H&E; 400x); **B –** CD3 showing diffuse membranous positivity (peroxidase; 100x); **C –** CD20 is negative (peroxidase; 100x); **D –** Diffuse membranous positivity of CD56 (peroxidase; 400x).

**Figure 2:**
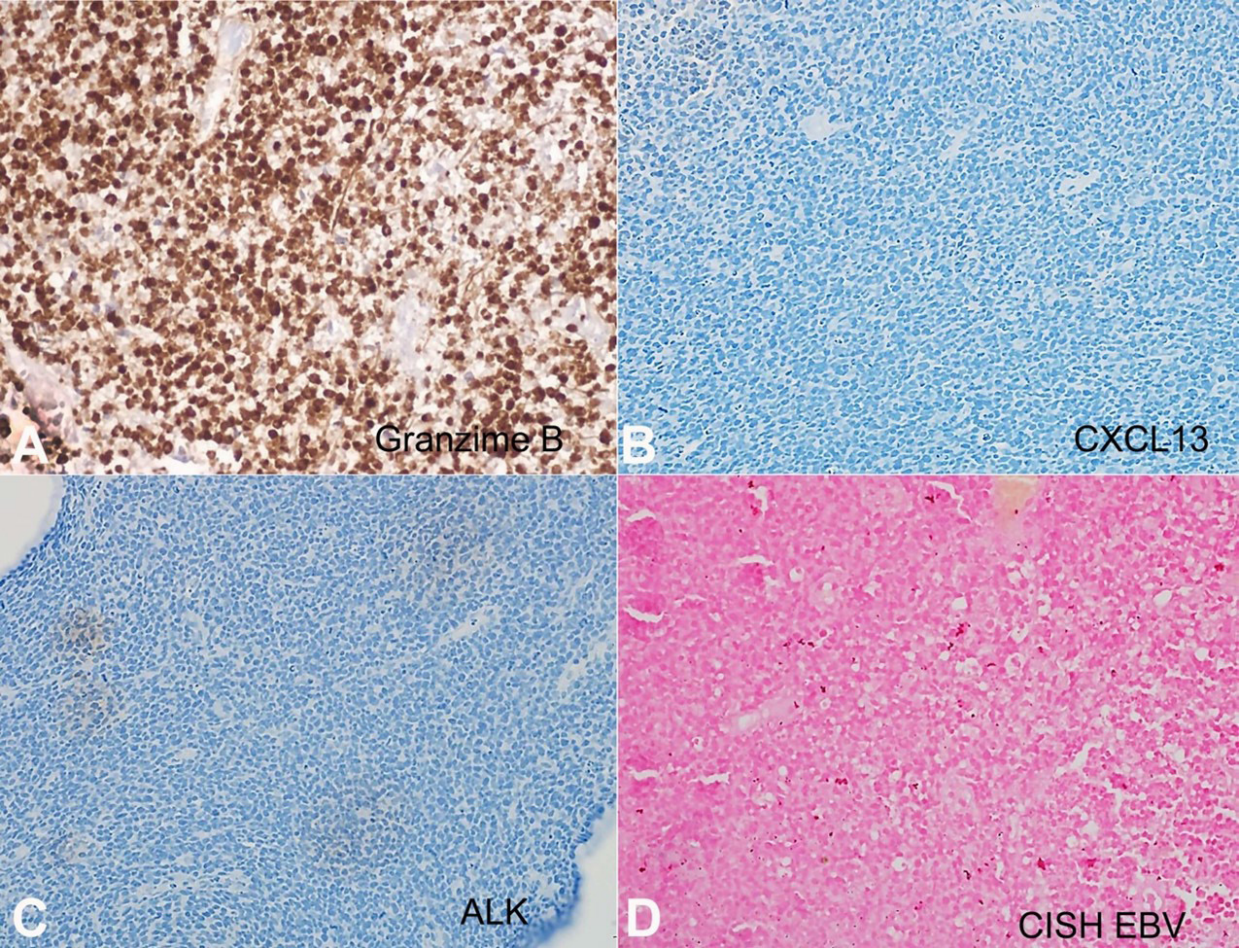
Photomicrographs of the cervical tumor. **A –** Granzyme B showing diffuse cytoplasmic granular positivity (peroxidase; 400x); **B –** CXCL13 is negative (peroxidase; 400x); **C –** ALK is negative (peroxidase; 400x); **D –** Chromogenic in-situ hybridization (CISH) for EBV-encoded RNA (EBER-1) (Ventana) was negative (positive external control was present; image not shown).

**Figure 3:**
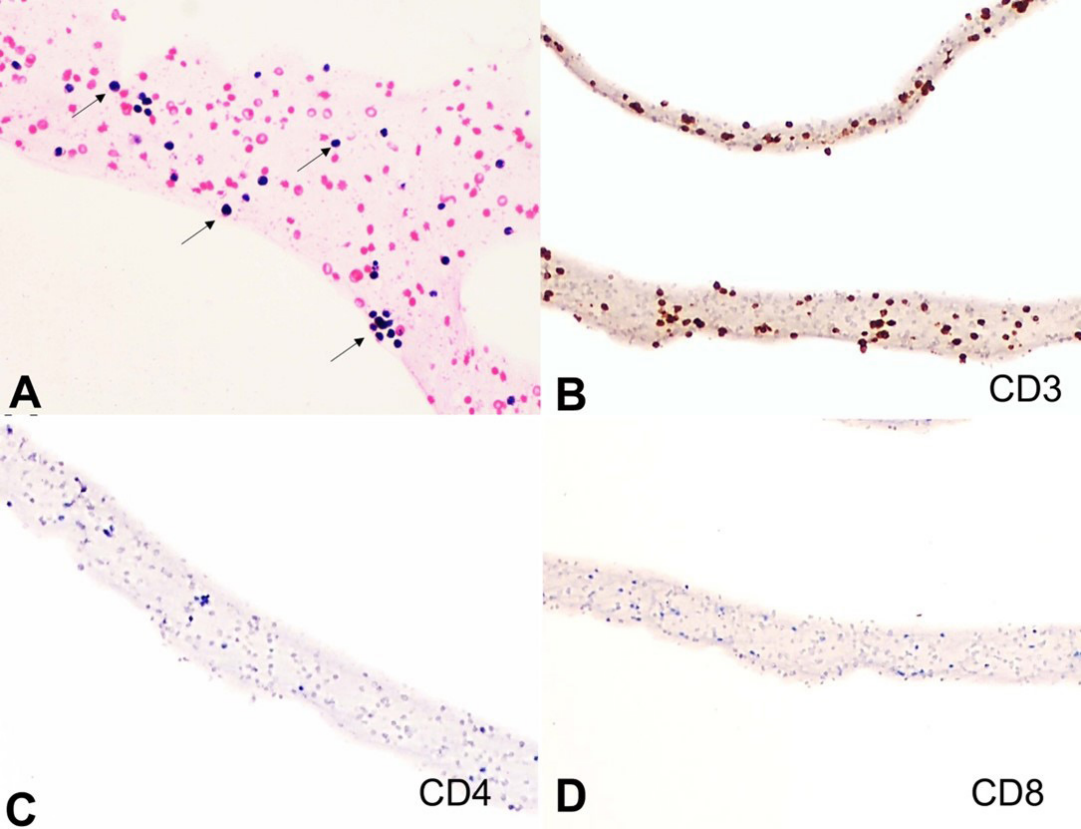
Cell block preparation from CSF cytology and limited panel of immunohistochemistry. **A –** Scattered atypical small-sized lymphoid cells (black arrows) (H&E; 200x); **B –** showing diffuse CD3 positivity (200x); **C –** These cells exhibit CD4 (200x); **D –** CD8 (200x) negativity.

Grossly, the polypoidal cervical biopsy measured 4.4x3.2x2.7cm. The mucosal aspect was smooth, whereas the cut surface appeared firm and homogeneous. Microscopically, the subepithelium of the cervix was completely effaced by diffuse dyscohesive collection of the atypical lymphoid cells. These atypical lymphoid cells were small-to-medium sized and had a high N:C ratio, angulated nuclear membrane, opened-up chromatin, inconspicuous nucleoli, and scant amount of cytoplasm (Figure 1A). Brisk mitosis, numerous apoptotic bodies, and foci of necrosis were seen. These cells were positive for LCA (diffuse strong membranous), and negative for pancytokeratin, desmin, CD10, and synaptophysin. Further, these cells were positive for surface CD3 (Figure 1B) and negative for CD20 (Figure 1C) and PAX5. CD56 exhibited diffuse, moderate to strong membranous positivity (Figure 1D). CD5 (patchy, variable-intensity, membranous), granzyme B (diffuse strong granular cytoplasmic) (Figure 2A), and Bcl2 (diffuse strong cytoplasmic) were positive, whereas TdT, CD4, CD8, CD7, CD57, PD-1, CXCL13 (Figure 2B), Bcl6, GATA3, CD30, and ALK (Figure 2C) were negative. Chromogenic in-situ hybridization (CISH) for EBV-encoded RNA (EBER-1) (Ventana) was negative (positive external control was present) (Figure 2D). Ki-67 index was 85-90%. A diagnosis of extranodal peripheral T-cell lymphoma, not otherwise specified (extranodal PTCL, NOS) was rendered. Cell block prepared from CSF yielded low cellularity with few scattered small-sized lymphoid cells showing condensed chromatin (Figure 3A). These cells were positive for CD3 (Figure 3B) whereas negative for both CD4 (Figure 3C) and CD8 (Figure 3D). Further, IHC panel could not be performed due to tumor cell exhaustion. Considering the morphology and immunohistochemistry (CD3+/CD4-/CD8-) findings, the possibility of CSF infiltration by the lymphoma cells was kept.

The patient was immediately started on a combination of cyclophosphamide, etoposide, and steroids, but the patient deteriorated rapidly and succumbed to the illness despite our best efforts with hardly one week of follow-up. The cause of death was uncertain, and the autopsy could not be performed as the permission was denied. However, the most possible cause of the demise could be terminal septicemia.

## DISCUSSION

The incidence of primary gynecologic tract lymphoma is very low, accounting for 0.2% to 1.1% of all cases of extranodal lymphoma.^7^ The challenge exists between primary and secondary lymphoma, especially when there is a nodal and extranodal disease. Most of the non-Hodgkin lymphomas (NHLs) in the female genital tract (FGT) involve the ovaries, commonly as a part of a disseminated form (7-30%).^7^ Diffuse large B-cell lymphoma is the most common histological subtype.^3^^,^ 8 In a large series encompassing 129 cases of primary NHL of the cervix, only 3.1% of the cases were diagnosed as T-cell NHL.^3^ Peripheral T-cell lymphoma (PTCL) is a clinically aggressive disease, with a poor response to therapy and 5-year overall survival (OS) of only 30%, approximately.^9^ PTCL, involving primarily the cervix, is very uncommon, and the English literature contains solo case report.^2^ The index case presented with uncommon primary PTCL, NOS of the cervix, and an unusual association of peripheral neuropathy.

Mononeuritis multiplex is a form of peripheral neuropathy characterized by painful, asymmetric, asynchronous involvement of two or more sensory/ motor nerves that are non-adjacent. The common causes of mononeuritis multiplex are leprosy, vasculitic disorders mainly polyarteritis nodosa, autoimmune disorders like systemic lupus erythematosus and rheumatoid arthritis, diabetes mellitus, human immunodeficiency virus, sarcoidosis, amyloidosis, and paraneoplastic.^10^^,^ 11 Approximately 8% of cases of mononeuritis multiplex are associated with hematological malignancy, the majority of which are B-cell NHL.^6^ The occurrence of peripheral neuropathy/ mononeuritis multiplex associated with T-cell NHL/ PTCL, NOS is uncommon and is documented in fewer patients.^5^^,^ 6 Neuropathy associated with lymphoma can be classified broadly into neurolymphomatosis, which represents the direct invasion of lymphoma cells into the peripheral nervous system, and paraneoplastic neuropathy, which represents damage remote from the site of lymphoma.^12^ The evidence of neurolymphomatosis is usually detected on FDG-PET study and confirmed on biopsy or autopsy. In the case of paraneoplastic neuropathy, the pathological findings suggestive of chronic inflammatory demyelinating polyneuropathy (CIDP), sensory ganglionopathy, or vasculitis neuropathy can be seen.^6^ None of these mechanisms were confirmed in the index patient due to the non-availability of consent to perform the nerve biopsy or autopsy.

Histopathologically, these tumors show variable cell morphology (small-to-medium-to-large) and express pan–T-cell–associated antigens.^1^ A CD4+/CD8- phenotype is ideally seen in PTCL, NOS, although double negativity (CD4-/CD8-) is sometimes noted.^13^ The index case exhibited CD4-/CD8- phenotype, but then strong and diffuse CD56 expression prompted us to rethink the diagnosis. In PTCL, CD56 expression is unusual in nodal PTCL cases; however, it can be seen in extranodal sites.^14^ The likelihood of extranodal NK/T-cell lymphoma, the nasal type was ruled out in view of the EBV-encoded RNA (EBER-1) negativity on chromogenic in-situ hybridization (CISH). The negativity for CD30 and ALK exclude the possibility of both ALK+ and ALK- anaplastic large cell lymphoma (ALCL), while the negativity for CD20 and PAX5 rules out a B-cell NHL. Besides, a possibility of lymphoblastic lymphoma/ leukemia is negated by the TdT negativity. We also looked for the T follicular helper (TFH) cell phenotype, but CXCL13, PD1, CD10 and Bcl6 were negative. A primary immunopanel consisting of pancytokeratin, synaptophysin, CD99, desmin, and CD10 was required to rule out the possibilities of other small blue round cell tumors with similar histomorphology namely metastatic small cell carcinoma, primitive neuroectodermal tumors/ Ewing sarcoma, rhabdomyosarcoma, and endometrial stromal sarcoma. Due to the rarity of PTCL, NOS at the extranodal sites, the specific treatment protocol is difficult to decide. The index patient was started on a cycle of chemotherapy regime, but she could not revive and succumb to her illness within one week.

## CONCLUSION

The primary peripheral T-cell lymphoma of the uterine cervix is extremely rare and therefore, there are no specific management guidelines to decide. Nerve biopsy or autopsy is needed to rule out the cause for the associated neuropathy, which could not be performed in our patient because of the non-availability of the consent. Both the physicians and the pathologists need to be aware of such rarity. They need to keep a high level of suspicion while dealing with the protean manifestations of the lymphoid malignancies.
